# Corneal differences between healthy and subclinical patients assessed using two different corneal tomographers

**DOI:** 10.5935/0004-2749.20200015

**Published:** 2020

**Authors:** Haixia Zhao, Zhaoping Yang, Xiaotong Han, Wenying Guan, Zhaoge Wang, Meilan Cai, Yi Sun, Ruichun Ge, Ruifang Wang

**Affiliations:** 1 Department of Ophthalmology, The Affiliated Hospital of Inner Mongolia Medical University, Huhhot 010050, China

**Keywords:** Keratoconus/diagnosis, Corneal topography, Cornea/diagnostic imaging, Diagnostic imaging/methods, Com parative study, Ceratocone/diagnóstico, Topografia da córnea, Córnea/diagnóstico por imagem, Diagnóstico por imagem/métodos, Estudo comparativo

## Abstract

**Purpose:**

To analyze subclinical keratoconus topography indexes using Pentacam and Orbscan-II measurements to identify evidences for seeking sensitive indexes to screen and diagnose subclinical keratoconus.

**Methods:**

Fifty healthy participants (50 eyes) and 40 patients with subclinical keratoconus (40 eyes) were included. Seven common parameters including corneal thickness at the thinnest point; minimum curvature of the front surface (minimum simulated keratometry value, SimK’s Min); maximum curvature of the front surface (maximum simulated keratometry value, SimK’s Max); the frontal corneal surface best-fit spherical radius of the curvature; the back corneal surface best-fit spherical radius of curvature; the anterior corneal surface height (anterior Diff value); and the posterior corneal surface height (posterior Diff value) measured by Pentacam and Orbscan-II between normal and subclinical keratoconus eyes were compared.

**Results:**

Statistical differences between the healthy and subclinical keratoconus groups (p<0.01) were found in all corneal parameters measured using both devices. Differences in the minimum curvature of the front surface (SimK’s Min), thinnest point, anterior Diff value, and posterior Diff value were significant between Pentacam and Orbscan-II in the subclinical keratoconus group (p<0.05).

**Conclusion:**

The findings of this study identify the differences between normal and subclinical keratoconus eyes at the minimum curvature of the front surface, maximum curvature of the front surface, frontal corneal surface best-fit spherical radius of curvature, back corneal surface best-fit spherical radius of curvature, Anterior Diff value, and Posterior Diff value measures using Orbscan II and Pentacam that can help eye care practitioners clinically diagnose subclinical keratoconus.

## Abstract (pt)

**Objetivo:**

Analisar os índices subclínicos de to pografia de ceratocone utilizando as medidas feitas com Pentacam e com Orbscan-II para identificar evidências para a busca de índices sensíveis para triagem e diagnóstico de ceratocone subclínico.

**Métodos:**

Cinquenta participantes saudáveis (50 olhos) e 40 pacientes com ceratocone subclínico (40 olhos) foram incluídos. Sete parâmetros comuns, incluindo a espessura da córnea no ponto mais fino; a curvatura mínima da superfície frontal (valor mínimo da ceratometria simulada, Min de SimK); a curvatura máxima da superfície frontal (valor máximo da ceratometria simulada, Max de SimK); a superfície frontal e a superfície posterior da córnea de melhor ajuste ao raio da curvatura, a altura da superfície anterior da córnea (valor Diff anterior) e a altura da superfície corneana posterior (valor Diff posterior) medidos pelo Pentacam e pelo Orbscan-II entre os olhos normais e com ceratocone subclínico foram comparados.

**Resultados:**

As diferenças estatísticas entre os grupos saudável e com ceratocone subclínico (p<0,01) foram encontradas em todos os parâmetros corneanos medidos usando ambos os dispositivos. Diferenças na curvatura mínima da superfície frontal (Min de SimK) no ponto mais fino, no valor Diff anterior e no valor Diff posterior foram significativas entre Pentacam e Orbscan-II no grupo com ceratocone subclínico (p<0,05).

**Conclusão:**

Os achados deste estudo identificam as diferenças entre olhos normais e com ceratocone subclínico para a curvatura mínima da superfície frontal, a curvatura máxima da superfície frontal, a superfície corneana frontal e a superfície corneana posterior de melhor ajuste ao raio esférico da curvatura e as medidas de Diff anterior e posterior usando Orbscan II e o Pentacam que podem auxiliar os profissionais de oftalmologia a diagnosticar clinicamente o ceratocone subclínico.

## INTRODUCTION

Keratoconus is a progressive corneal disorder characterized by central and paracentral cornea steepening that is an absolute contraindication for corneal refractive surgery^(1)^. This corneal denaturation disease commonly occurs in patients aged >40 years. A few pa tients may develop serious complications, such as corneal cicatrix or even perforation^(2)^. A study has shown that refractive surgery may stimulate occult keratoconus and turn it into an active phase, which can become an iatrogenic keratoconus^(3)^. Therefore, subclinical keratoconus that have no biomicroscopic clinical signs should be screened and diagnosed via a careful corneal topography analysis involving the posterior corneal curvature to avoid iatrogenic corneal ectasia after a refractive surgery^(4-6)^. Nowadays, the main methods used for screening keratoconus are corneal tomography using different devices, such as Orbscan-II tomographer (based on slit-scan) and Pentacam (based on Scheimpflug principle)^(7)^. Therefore, this prospective study was conducted to compare the seven common corneal parameters measured with Orbscan-II and Pentacam in normal and subclinical keratoconus eyes, in order to identify evidences to establish sensitive clinical criteria to screen and diagnose subclinical keratoconus.

## METHODS

### Subjects

Ninety participants with refractive errors at the myopia cure center of Inner Mongolia Medical University Affiliated Hospital from July 2011 to September 2015 were enrolled in this study. The inclusion criteria were as follows: i) intraocular pressure (IOP) of 10-21 mmHg, examined using a slit lamp to ensure the absence of corneal scar that may affect IOP measurement; ii) patients with monocular subclinical keratoconus; iii) those who wear soft contact lenses that were discontinued more than 2 weeks and rigid contact lenses that were discontinued for a minimum of 4 weeks. The exclusion criteria were as follows: i) history of ocular trauma, surgery, chronic inflammation, corneal diseases except keratoconus, and family history of glaucoma; and ii) connective tissue diseases, autoimmune diseases, and other systemic diseases. According to the Rabinowitz diagnostic criteria (the disease did not meet the diagnostic criteria of clinical keratoconus, but conformed to the following criteria: i) central corneal refraction of >46.5 D; <3 mm and >1.26 D difference of the corneal curvature; and iii) >0.92 D) corneal refractive difference of double eyes from the same patient^(8)^. A total of 90 patients were divided into normal (50 patients with 50 eyes; receiving refractive surgery) and subclinical keratoconus group (40 patients with 40 eyes). This study was approved by the ethics committee of Inner Mongolia Medical University. Written informed consent was obtained from all participants.

### Pentacam assessment

Pentacam and Obscan assessments were performed on the same day, with >10-min interval to allow relaxation and tear film recovery. Pentacam assessment was performed in a darkroom, under a natural pupil condition. The patient was instructed to sit in front of the instrument, with the lower jaw on the tray. The patient watched the flashing blue light in front, try to open eyes to expose the entire cornea and not blink. The examiner moved the Pentacam by operating the handle to adjust the arrow on the display. When the pupil coincided with the corneal center and the focus coincided with the corneal vertex, images were collected using the instrument. The test data could be used only when the image quality was acceptable and the quality factor was >95%. In order to reduce measurement error, all tests per eye were checked at least three times by the same experienced operator to select the best repeatability.

### Orbscan-II assessment

Orbscan topography was also performed in a darkroom. The lower jaw was placed on the tray, with forehead close to the band, and the head fixed by a band. The patient was asked to focus on the flashing red light ahead and try to open the eyes to expose the whole cornea. The examiner moved the handle and adjusted the focus of the optical head, to determine whether the cornea was placed at the center of the monitor. When two half-slit lights mapped on the cornea coincided like an “S” type, the patient was instructed to stare at the red light and not blink. Images were collected by the instrument, and 3D images were reconstructed. In order to reduce measurement error, all tests per eye were checked at least three times by the same experienced operator and those with the best repeatability were selected. In the measurement process, acquired images with poor clarity due to ocular surface dryness, blinking, eye movement, and poor cornea exposure were rejected, and corneal topography was repeated to guarantee capturing of high-quality images.

### Detection parameters

Seven Pentacam and Orbscan-II outcomes were collected: Corneal thickness of the thinnest point (TP), minimum curvature of the front surface (K1 Pentacam and SimK’s Min Orbscan), maximum curvature of the front surface (K2 Pentacan and SimK’s Max Orbscan), frontal corneal surface best-fit spherical radius of the curvature (FBFS), back corneal surface best-fit spherical radius of curvature (BBFS), anterior corneal surface height (anterior Diff value), and posterior corneal surface height (posterior Diff value).

### Statistical analysis

SPSS 17.0 (SPSS, Chicago, IL, USA) statistical package for Windows was used for statistical analysis. Seven corneal outcomes with each measurement between the normal control and subclinical keratoconus groups were compared, and seven corneal outcomes in each group between Pentacam and Orbscan-II measurements were also compared. Shapiro-Wilk test was used for the normal distribution data, and rank sum test for the non-normally distribution data. The t-test was used for parameter data, and the rank sum test was used for non-parametric data. P<0.05 was considered statistically significant.

## RESULTS

### Patients’ general information

Among the 90 patients, 49 were males and 41 females, with the mean age of 23.7 ± 0.7 (range, 17-40) years. The normal group consisted of 25 males and 25 females, with the mean age of 22.8 ± 0.9 years. No abnormal signs were observed in the slit lamp and corneal topography tests, spherical equivalent <-3.0 D, astigmatism <-1.0 D, and best-corrected visual acuity ≥1.0 (Snellen standard chart). The subclinical keratoconus group consisted of 24 males and 16 females, with the mean age of 23.4 ± 1.7 years. No obvious signs of keratoconus were detected after the slit lamp and ophthalmoscopy examination, and the best-corrected visual acuity was performed using the correcCorneal topography showing the following results: i) central corneal refractive of >46.5 D; ii) <3 mm and >1.26 D difference of the corneal curvature; and iii) >0.92 D corneal refractive difference of double eyes from the same patient.

### Comparison of Pentacam parameters between the two groups

When comparing the Pentacam parameters between two groups, the TP, FBFS, and BBFS in the normal group were significantly higher than those in the subclinical keratoconus group (p 0.01). However, K2, K1, anterior Diff value, and posterior Diff value in the subclinical keratoconus group were significantly higher than those in the normal group (all p<0.01) (Table 1).

**Table 1:** Comparison of Pentacam parameters between the two groups (median (interquartile range))

Group	Eyes	TP (µm)	K1 (D)	K2 (D)	ABFS (mm)	PBFS (mm)	Anterior Diff value (µm)	Posterior Diff value (µm)
Normal	50	543.00 (27.00)	42.48 (1.76)	43.31 (2.20)	7.94 (0.34)	6.45 (0.23)	6.51 (3.00)	16.00 (8.00)
Subclinical keratoconus	40	478.00 (25.00)	44.05 (3.13)	45.35 (4.16)	7.53 (0.51)	6.10 (0.45)	13.00 (5.55)	25.50 (4.76)
Z		44.771	29.085	48.614	37.155	18.644	63.308	64.966
P		0.002	0.005	0.001	0.004	0.004	0.002	0.001

TP= thinnest point; K1= minimum curvature of front surface; K2= maximum curvature of front surface; ABFS= anterior corneal surface best-fit spherical radius of curvature; PBFS= posterior corneal surface best-fit spherical radius of curvature; anterior Diff value, anterior corneal surface height; posterior Diff value, posterior corneal surface height. Z= Z test; P= probability.

### Comparison of Orbscan-II parameters between the two groups

The Orbscan-II parameters of the two groups are showed in table 2. TP and BBFS in the normal group were significantly higher than those in the subclinical keratoconus group (p<0.01). However, SimK’s Min (K1), SimK’s Max (K2), anterior Diff value, and posterior Diff value in the subclinical keratoconus group were significantly higher than those in the normal group (all p<0.01). No significant difference was observed in FBFS between the two groups (p>0.05).

**Table 2:** Comparison of Orbscan-II parameters between the two groups (median (interquartile range))

Group	eyes	TP (µm)	Sim’s Min (D)	Sim’s Max (D)	ABFS (mm)	PBFS (mm)	Anterior Diff value (µm)	Posterior Diff value (µm)
Normal	50	549.00 (53.00)	41.95 (1.60)	43.30 (1.98)	7.95 (0.28)	6.43 (0.27)	16.00 (9.00)	31.00 (13.00)
Subclinical keratoconus	40	495.00 (15.00)	42.34 (3.55)	44.30 (4.02)	7.55 (0.48)	6.24 (0.41)	24.00 (9.30)	46.00 (13.00)
Z		33.627	11.690	13.980	33.560	19.853	31.295	42.971
P		0.001	0.002	0.001	0.155	0.002	0.002	0.001

TP= thinnest point; SimK’s Min= minimum simulated keratometry value; SimK’s Max= maximum simulated keratometry value; ABFS= anterior corneal surface best-fit spherical radius of curvature; PBFS= posterior corneal surface best-fit spherical radius of curvature; anterior Diff value, anterior corneal surface height; posterior Diff value, posterior corneal surface height. Z= Z test; P= probability.

### Comparison of Pentacam and Orbscan-II outcomes in the normal group

K1 in the Pentacam measurement was significantly higher than that in Orbscan-II measurement (U=-2.980, p<0.01). The anterior and posterior Diff values in Orbscan-II measurement were significantly higher than those in the Pentacam measurement (U=49.487, p<0.01; U=53.672, p<0.01). Differences in other parameters between the Pentacam and Orbscan-II measurement were not statistically significant (p>0.05) (Table 3).

**Table 3:** Comparison of basic parameters measured by Pentacam and Orbscan-II in the normal group (median (interquartile range))

Group	Eyes	TP (µm)	K1/Sim’s Min (D)	K2/Sim’s Max (D)	ABFS (mm)	PBFS(mm)	Anterior Diff value (µm)	Posterior Diff value (µm)
Pentacam	50	543.00 (27.00)	42.48 (1.76)	43.31 (2.20)	7.94 (0.34)	6.45 (0.23)	6.51 (3.00)	16.00 (8.00)
Orbscan-II	50	549.00 (53.00)	41.95 (1.60)	43.30 (1.98)	7.95 (0.28)	6.43 (0.27)	16.00 (9.00)	31.00 (13.00)
U		-0.963	-2.980	-0.002	0.008	-0.021	49.487	53.672
P		0.721	0.002	0.567	0.224	0.408	0.002	0.003

TP= thinnest point; K1= minimum curvature of front surface; K2, maximum curvature of front surface; SimK’s Min= minimum simulated keratometry value; SimK’s Max= maximum simulated keratometry value; ABFS, anterior corneal surface best-fit spherical radius of curvature; PBFS= posterior corneal surface best-fit spherical radius of curvature; anterior Diff value, anterior corneal surface height; posterior Diff value, posterior corneal surface height. U= U test; P= probability.

### Comparison of Pentacam and Orbscan-II outcomes in the subclinical keratoconus group

Pentacam provides significantly higher K1 values than Orbscan-II (U=8.208, p=0.032) in the subclinical keratoconus group; however, the TP, anterior Diff value, and posterior Diff value were significantly lower than those achieved with Orbscan (p<0.05). Differences in the remaining parameters between Pentacam and Orbscan-II measurement were not statistically significant (p>0.05) (Table 4).

**Table 4:** Comparison of basic parameters measured by Pentacam and Orbscan-II in the subclinical keratoconus group (median (interquartile range))

Group	Eyes	TP (µm)	K1/Sim’s Min (D)	K^^2^^/Sim’s Max (D)	ABFS (mm)	PBFS (mm)	Anterior Diff value (µm)	Posterior Diff value (µm)
Pentacam	40	478.00 (25.00)	44.05 (3.13)	45.35 (4.16)	7.53 (0.51)	6.10 (0.45)	13.00 (5.55)	25.50 (4.76)
Orbscan-II	40	495.00 (15.00)	42.34 (3.55)	44.30 (4.02)	7.55 (0.48)	6.24 (0.41)	24.00 (9.30)	46.00 (13.00)
U		33.321	8.208	0.141	-0.244	-0.137	16.333	25.183
P		0.031	0.032	0.210	0.414	0.762	0.022	0.024

TP= thinnest point; K1= minimum curvature of front surface; K2= maximum curvature of front surface; SimK’s Min, minimum simulated keratometry value; SimK’s Max= maximum simulated keratometry value; ABFS= anterior corneal surface best-fit spherical radius of curvature; PBFS, posterior corneal surface best-fit spherical radius of curvature; anterior Diff value, anterior corneal surface height; posterior Diff value, posterior corneal surface height. U= U test; P= probability.

## DISCUSSION

Keratoconus is one of the developmental abnormal corneal diseases incidentally detected during a pre-refractive surgery assessment. In recent years, the rapid development of anterior segment analysis system has promoted continuous improvement in the clinical diagnostic skills^(9)^. Orbscan-II and Pentacam are the two devices that can not only analyze the anterior cornea surface qualitatively and quantitatively but can also measure the information in the posterior surface. Therefore, sensitive indicators to screen subclinical keratoconus are necessary to provide theoretical basis for its early diagnosis and to provide a more comprehensive reference for preoperative screening and postoperative evaluation of corneal refractive surgery.

Our results are consistent with those of previous reports that compared Orbscan-II and Pentacam to screen, diagnose, and evaluate keratoconus, finding differences between both devices suggesting that they are not interchangeable^(10,11)^. Subclinical keratoconus diagnostic criteria with Orbscan-II have been previously described as follows: the anterior Diff value of ≥0.025; back Diff value of ≥0.050; SimK (difference between SimK’s Max and SimK’s Min in one eye) of ≥4.5 D; corneal refractive power of >46.5 D; and TP of ≤460 µm corneal thickness^(12)^. Souza et al.^(13)^ used support vector machines, multilayer perceptron, and radial basis function neural network measured by Orbscan-II to effectively diagnose keratoconus. Moreover, other scholars also distinguish normal cornea and expanded cornea using the Pentacam^(14)^.

Greenstein et al.^(15)^ have speculated that the pathogenesis of keratoconus is associated with increased colla genase and metalloproteinase activity, decreased corneal stromal collagen tissue, and inadequate corneal tissue to resist the normal intraocular pressure and thus resulting in bulging forward. Therefore, as the first barrier against increased intraocular pressure, changes in the posterior corneal surface caused by the IOP is more obvious. The excimer laser myopia operation principle is used to ablate corneal thickness, even decreasing the normal corneal strength, and if the patient has subclinical keratoconus, more likely develops into severe iatrogenic keratoconus^(16)^. Posterior surface height is very important in screening subclinical keratoconus; therefore, many scholars dedicated on the study of posterior corneal surface^(4,5)^. Nilforoushan et al.^(17)^ thought that Pentacam has a higher posterior Diff value and is associated with thinner cornea, and Orbscan-II has higher values in anterior and posterior surface height, with the thinnest corneal point displaced to the side of the nose.

By comparing the corneal posterior surface height measured by Pentacam and Orbscan-II, values obtained with Pentacam are found to be smaller, which is consistent with Núñez and Blanco’s study^(18)^. This may occur because of different principles used to capture corneal topography because Orbscan-II uses a combination of fracture scanning and Placido technology, whereas Pentacam uses rotating scanning principle, which can focus in the deep view and clearly show the anterior and posterior corneal surface images; therefore, the veracity of Pentacam is higher when measuring the corneal surface, especially the posterior surface^(7)^.

The thinnest corneal thickness, anterior Diff value, and posterior Diff value are three parameters most widely accepted as sensitive topographic map indicators; however, this study also found that FBFS and BBFS have significant differences between the normal and subclinical keratoconus groups; therefore, FBFS and BBFS are thought to be likely sensitive to the topographic index; however, their diagnostic value is smaller than the three parameters above. The number of males is higher in patients with subclinical keratoconus in this study, which is consistent with a previous report^(19)^. As the number of patients included in this study is small, further research to expand the sample size with cut-off value of receiver operating characteristic curve will be necessary in prospective studies.

The characteristic stromal thinning in keratoconus corresponds to an increased posterior elevation above the best-fit sphere in corneal topography. In Hashemi and Mehravaran’s study on myopic patients who underwent laser in situ keratomileusis (LASIK) or photorefractive keratectomy (PRK), interdevice differences were statistically significant for anterior chamber depth, anterior corneal axial power, and all posterior corneal parameters. Topography of the anterior corneal surface forms the basis of all ectasia detection indices and scores. Recently, topography and biomechanical assessment were used for the detection of corneas that may be predisposed to ectasia. Some of these eyes with ectasia may possibly have subclinical keratoconus preoperatively and thereby indications were missed. Thus, these eyes could have naturally progressed to manifest keratoconus in the following years or surgery could have led to progression. In conclusion, this study shows differences between normal and subclinical keratoconus eyes in K1, K2, FBFS, BBFS, anterior Diff value, and posterior Diff value measures with Orbscan-II and Pentacam that could help eye care practitioners in the clinical diagnosis of subclinical keratoconus.
